# A Synthetic Strong and Constitutive Promoter Derived from the *Stellaria media* pro-SmAMP1 and pro-SmAMP2 Promoters for Effective Transgene Expression in Plants

**DOI:** 10.3390/genes11121407

**Published:** 2020-11-26

**Authors:** Larisa N. Efremova, Svetlana R. Strelnikova, Guzel R. Gazizova, Elena A. Minkina, Roman A. Komakhin

**Affiliations:** 1 All-Russia Research Institute of Agricultural Biotechnology, Moscow 127550, Russia; laraefremova@mail.ru (L.N.E.); strlnkv@yandex.ru (S.R.S.); 2 Institute of Fundamental Medicine and Biology, Kazan Federal University, Kazan 420008, Russia; grgazizova@gmail.com (G.R.G.); minkinaea@gmail.com (E.A.M.)

**Keywords:** synthetic promoter, GUS, ACTCAT cis-element, *nptII*, genetic transformation, gene expression

## Abstract

Synthetic promoters are vital for genetic engineering-based strategies for crop improvement, but effective methodologies for their creation and systematic testing are lacking. We report here on the comparative analysis of the promoters pro-SmAMP1 and pro-SmAMP2 from *Stellaria media ANTIMICROBIAL PEPTIDE1* (*AMP1*) and *ANTIMICROBIAL PEPTIDE2* (*AMP2*). These promoters are more effective than the well-known *Cauliflower mosaic virus* 35S promoter. Although these promoters share about 94% identity, the pro-SmAMP1 promoter demonstrated stronger transient expression of a reporter gene in *Agrobacterium* infiltration of *Nicotiana benthamiana* leaves, while the pro-SmAMP2 promoter was more effective for the selection of transgenic tobacco (*Nicotiana tabacum*) cells when driving a selectable marker. Using the cap analysis of gene expression method, we detected no differences in the structure of the transcription start sites for either promoter in transgenic plants. For both promoters, we used fine-scale deletion analysis to identify 160 bp-long sequences that retain the unique properties of each promoter. With the use of chimeric promoters and directed mutagenesis, we demonstrated that the superiority of the pro-SmAMP1 promoter for *Agrobacterium*-mediated infiltration is caused by the proline-inducible ACTCAT *cis*-element strictly positioned relative to the TATA box in the core promoter. Surprisingly, the ACTCAT *cis*-element not only activated but also suppressed the efficiency of the pro-SmAMP1 promoter under proline stress. The absence of the ACTCAT *cis*-element and CAANNNNATC motif (negative regulator) in the pro-SmAMP2 promoter provided a more constitutive gene expression profile and better selection of transgenic cells on selective medium. We created a new synthetic promoter that enjoys high effectiveness both in transient expression and in selection of transgenic cells. Intact promoters with differing properties and high degrees of sequence identity may thus be used as a basis for the creation of new synthetic promoters for precise and coordinated gene expression.

## 1. Introduction

The analysis of promoters is essential for the elucidation of coordinated gene expression in plant cells under changing environmental conditions. The native “full-length” promoters of protein-coding genes consist of a distal, a proximal, and a core promoter region. The core promoter contains the TATA box regulatory sequence, located about 30–40 base pairs (bp) upstream of the transcription start site (TSS) where the core transcriptional machinery (RNA polymerase II (RNA PolII), TATA-binding proteins (TBPs) and TBP-associated factors) interact to initiate transcription [1]. TATA-type core promoters only account for 20–30% of plant promoters [2,3,4]; TATA-less core promoters have not been subjected to a comprehensive experimental characterization [5]. The physical interaction between the core transcriptional machinery and regulatory proteins in the promoter proximal region is determined during the initiation of transcription by proximal elements adjacent to the core promoter. Distal promoter elements also influence gene expression due to DNA folding-induced conformational changes in the 3-dimensional structure of DNA and the surrounding chromatin [1].

Various DNA regulatory sequence elements such as enhancers, silencers, insulators, and *cis*-elements are distributed across promoter regions and contribute to the functional architecture of a “full-length” promoter [6]. Crosstalk between different regulatory sequences in the promoter and different protein factors play major roles in imparting tissue specificity and expression strength in eukaryotic promoters. At present, the complexity and organization of plant promoters do not allow in silico predictions of the expression pattern of a given gene based on the specific regulatory elements identified within its promoter. However, a number of databases do exist that gather such information in very broad terms [6,7,8]. Various and partially overlapping regulatory sequences are combined within the context of a native “full-length” promoter, and the hierarchical integration of these elements by the plant transcriptional network is currently unknown. The applicability of such promoters in plant biotechnology for the precise transcriptional regulation of transgenes is therefore limited.

It is generally accepted that native promoters from dicotyledonous plants are typically much weaker than the strong and constitutive viral promoters (e.g., *Cauliflower mosaic virus* 35S promoter (CaMV35S), MUASMSCP derived from *Mirabilis mosaic virus*, and a promoter from *Figwort mosaic virus* (FMV)) that have been commonly exploited in plant biotechnology ([9,10,11] and other works). However, promoters isolated from plant pathogens may lead to abnormal phenotypes in transgenic plants [8]. In addition, these promoters are less preferable from an ecological point of view and may cause regulatory concerns due to the presence of viral sequences. The activity of virus-based promoters may also become suppressed during infection of genetically modified plants by viruses [12]. Currently, the promoters from dicotyledonous plants 400–500 bp upstream of the TSS are available. They offer great promise for use as strong and constitutive promoters in biotechnological applications (for example, [13,14,15,16,17,18,19]); however, how they control gene expression is largely unknown. 

The precise and coordinated expression of transgenes will require novel synthetic promoters with genetically programmable properties. Despite several methods aimed at constructing synthetic promoters (chimeric promoters [20], linker-scanning mutagenesis [21], DNA shuffling [22]), progress in this area has been hampered by the lack of experimentally characterized parameters for plant promoters, as well as by the absence of reliable methodologies for their testing and improvement. In the yeast *Pichia pastoris*, synthetic core promoters have been efficiently produced de novo based on computational analysis of the frequency of regulatory sequences and nucleosome positioning of several native promoters, followed by experimental validation [23]. However, similar approaches have largely been unsuccessful for plant biotechnology purposes [24]. At present, effective synthetic promoters based on the combination of multiple *cis*-elements or directed mutagenesis of *cis*-elements (such as G-box) have been created and validated experimentally [25,26,27]. Nonetheless, the CaMV35S core region is still the minimal constitutive promoter of choice in plant studies [25,26,27].

We have previously reported that the promoters from the chickweed (*Stellaria media*) *ANTIMICROBIAL PEPTIDE* (*AMP*) genes *SmAMP1* [16] and *SmAMP2* [17], although less than 450 bp in length, exhibited specific promoter activity up to three times that of the CaMV35S viral promoter in transient expression assays in *Nicotiana benthamiana* leaves as well as in homozygous transgenic tobacco (*Nicotiana tabacum*) lines [19]. Both promoters were at least as effective as the duplicated 2 × CaMV35S promoter for the selection of transgenic *Arabidopsis* (*Arabidopsis thaliana*) and tobacco plants, based on resistance to kanamycin conferred by *neomycin phosphotransferase II* (*nptII*) expression.

Although pro-SmAMP1 and pro-SmAMP2 are 94% identical along their length, in the current study, we show that they drive distinct expression patterns. In particular, pro-SmAMP1 expressed the *uidA* reporter gene twice as strongly, relative to pro-SmAMP2 in transient *N. benthamiana* assays, while pro-SmAMP2 facilitated the selection of three times as many transgenic shoots during the selection of transformed tobacco calli in the presence of excess (350 mg/L) kanamycin [19]. We capitalized on the high sequence identity but different responses of these promoters to define functional mutations or *cis*-elements that might be used to generate novel synthetic promoters featuring high and constitutive gene expression.

## 2. Materials and Methods

### 2.1. Plants for the Experiments

In the study plants of Bentham tobacco (*N. benthamiana* (Domin)), cultivated tobacco (*N. tabacum* L.), cultivar Samsun-NN, and *A. thaliana* L., ecotype Columbia-0, were used. The plants were grown in a 16-h light/8-h dark photoperiod and 22–24 °C unless specified. The illuminance of 70–80 μmol/m^2^ was used for cultivating aseptic plants and 150 μmol/m^2^ for greenhouse cultivation.

### 2.2. Novel Deletion, Chimeric and Synthetic Variants of Promoters

New deletion variants of pro-SmAMP1 and pro-SmAMP2 promoters were created with the PCR method using corresponding DNA matrices and primer pairs (Table A1 (Appendix A)). As the reverse primer, either Rev(amp1) or Rev(amp2) was used. Forward primers were denoted by the letter “F” with the corresponding length of the deletion variant. Deletion variants of the promoters comprised 5′-UTRs from the *SmAMP1* or *SmAMP2* genes. Nucleotide sequences of chimeric and synthetic promoters were synthesized by the PCR method with help of three partially overlapping primers. Due to primer sequences, all variants of promoters contained the restriction site *Eco*RI at the 5′-end and *Nco*I at the 3′-end. Initially they were cloned in pGEM T-vector (Promega, USA) and sequenced.

### 2.3. Genetic Constructs for Plant Transformation

For the construction of plasmids with the intron-containing *uidA* gene (pro-SmAMP1:*uidA* and pro-SmAMP2:*uidA*) under control of different promoters, corresponding nucleotide sequences were cut out from pGEM T-vector at restriction sites *Eco*RI and *Nco*I and ligated into the *Eco*RI-*Nco*I region of pCambia1381Z vector (Cambia, Australia). For the construction of plasmids with the *nptII* gene (pro-SmAMP1:*nptII* and pro-SmAMP2:*nptII*) under control of different promoters, corresponding nucleotide sequences were cut out from the pGEM T-vector at restriction sites *Eco*RI and *Nco*I and ligated into the pCambia2300 vector (Cambia, Australia) from which the nucleotide sequence of the 2 × CaMV35 duplicated viral promoter was removed at the same restriction sites.

### 2.4. Agrobacterium Strains

The GV3101 strain of *A. tumefaciens* was used for infiltration, whereas the AGL0 strain was used for genetic transformation. Following our previous research, in agroinfiltration in order to suppress the RNA interference, the GV2260/C58C1 agrobacterium strain with plasmid pLH7000 containing the p19 suppressor protein gene of tombusviruses was used [16].

### 2.5. Agrobacterium Infiltration of Plants

*Agrobacterium* infiltration of *N. benthamiana* leaves in every experiment was carried out at least thrice using 10 to 20 plants in each experiment for one construct. Detailed experimental conditions were described earlier [16].

### 2.6. Genetic Transformation of the Plants

*Agrobacterium* transformation of tobacco plants was carried out as described previously [28]. To evaluate efficiency of regeneration and selection of transgenic shoots with the use of each genetic construct, 10–14 leaf explants were analyzed; the experiment was performed thrice. Regeneration of shoots and calluses was evaluated during 2 to 3 months of cultivation on MS growth medium containing 350 or 550 mg/L of kanamycin antibiotic. *Agrobacterium* transformation of *Arabidopsis* plants was performed by the floral dip technique [29].

### 2.7. Proline Treatment

Aseptic transgenic tobacco T_1_ plants with monogenic T-DNA inheritance were cultivated for 4–6 weeks in 100 mL of Murashige and Skoog (MS) growth medium (with 30 g/L of sucrose and 0.8 g/L of agar). The treatment was carried out by adding 1.8 mL of sterile aqueous solution of proline L amino acid onto the surface of the growth medium (to reach the final concentration of 90 mM). For the plants from the control group, the same volume of sterile distilled water was added instead of proline solution. From each tobacco plant, the mid-shoot leaves were harvested in aseptic conditions before the treatment and on 1, 3, and 7 d after the treatment. The experiments with harvested leaves were carried out in accord with previously described conditions [30] with some modifications. Particularly, the leaves were cut along the midvein; one half of the leaf (control) was then dipped into the liquid MS medium (with no sucrose), and the other half was dipped either into liquid MS medium with 90 mM of proline or into liquid MS medium with 30 g/L of sucrose or into distilled water.

### 2.8. Quantitation of GUS Activity

Measurements of enzymatic activity of the GUS protein (resulting from *uidA* gene) in the plant extracts were performed using 4-methylumbelliferyl-β-D-glucuronide (PhytoTechnology Laboratories, Lenexa, KS, USA) as a substrate according to the method by Jefferson [31]. The detailed description of the employed technique was provided by us previously [16,17].

### 2.9. Analysis of Tobacco and Arabidopsis Plants with Polymerase Chain Reaction

For detection of *Agrobacterium* contamination the developed earlier, primers for the *VirE2* gene sequence were used [32]. For detection of the hybrid region of genetic construct containing the sequences of pro-SmAMP1 or pro-SmAMP2 promoters and of the *nptII* gene, the previously developed “olgminus” primer [19] and corresponding forward primers named with the letter “F” were used (Table A1).

### 2.10. RNA Extraction

Total RNA was extracted from plant leaves using Trizol reagent (Thermo Fisher Scientific, Waltham, MA, USA) according to the instructions of the manufacturer. To eliminate genomic DNA contamination, RNA was treated with DNase RQ1 RNase-Free (Promega, Madison, WI, USA) and stored at minus 70 °C. 

### 2.11. CAGE Library Preparation

Libraries were prepared according to single strand cap analysis of gene expression (SS-CAGE) protocol [33]. First, 5 µg of purified total RNA was used as a template for the synthesis of first strand cDNA (SS CAGE Library Preparation kit, DNAform, Yokohama, Japan and SuperScript III Reverse Transcriptase, Thermo Fisher Scientific, USA), which was then oxidized and biotinylated at the 5′-end (SS CAGE Library Preparation kit, DNAform). This made it possible to carry out 5′-cap trapping using streptavidin beads (Dynabeads M-270 Streptavidin, ThermoFisher Scientific, USA). At this step, RNA, which did not contain the 5′-cap (e.g., rRNA), as well as RNA, which was not completely reverse transcribed, were eliminated. For more efficient removal of nonspecific RNA strands, cDNA was treated with RNase I and H (SS CAGE Library Preparation kit, DNAform, Japan) and purified by RNACleanUP magnetic beads (Beckman Coulter, Brea, CA, USA). Next, linkers were ligated in sequence to the cDNA at the 5′and 3′ ends (SS CAGE Library Preparation kit, DNAform, Japan), and after that, libraries were cleaned up by AMPure XP magnetic beads (Beckman Coulter, USA). Then, the libraries were validated using real-time PCR (KAPA Library Quantification Kits Illumina, KAPA Biosystems, Wilmington, MA, USA), pooled, and sequenced on the HiSeq 2500 platform (Illumina, San Diego, CA, USA) using the HiSeq v4 reagent kit (HiSeq SR Cluster Kit v4 cBot and HiSeq SBS Kit v4 50 cycles, Illumina, USA) in single-ended 57 bp reads.

### 2.12. Statistical Data Processing

For statistical data processing, Student’s *t*-test in Microsoft Excel software was used. The mean values and the standard deviations are presented.

The alignment of nucleotide sequences of the promoters was performed by the Muscle algorithm in MEGA 7.0 software [34]. Promoter sequences were analyzed using PLACE [35] and PlantCARE [36] online tools.

## 3. Results

### 3.1. Mapping of the pro-SmAMP1 and pro-SmAMP2 Transcription Start Sites

**Figure 1:**
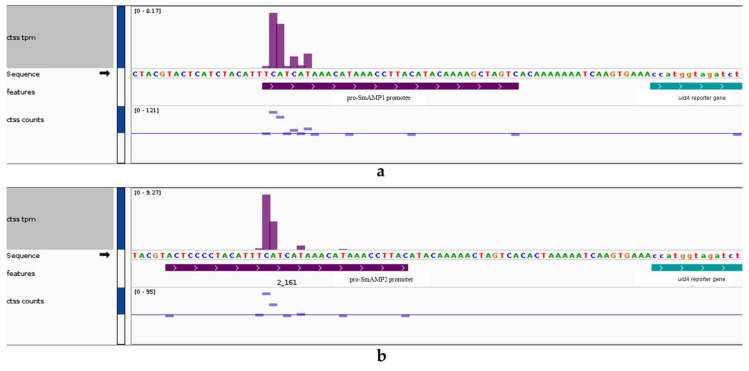
Cap analysis of gene expression data from pro-SmAMP1 (**a**) and pro-SmAMP2 (**b**) promoters in aseptic transgenic tobacco plants.

During our earlier characterization of pro-SmAMP1 and pro-SmAMP2 [16,17,19], we determined the position of the putative TSS from the cloning of the *SmAMP1* and *SmAMP2* genes from *S. media* [28]. Since the efficiency of transcription may depend on the TSS, we determined its position experimentally (Figure 1).

We performed cap analysis of gene expression (CAGE) using mRNA extracted from transgenic tobacco plants expressing the *uidA* reporter under the control of each pro-SmAMP promoter to precisely map TSSs. After trimming the adapters and removing low quality reads, we obtained 16,266,662 reads for pro-SmAMP1*:uidA* and 14,805,757 reads for pro-SmAMP2*:uidA*. For the pro-SmAMP1*:uidA* sample, 310 reads (0.002%) uniquely mapped to the pro-SmAMP1 promoter, forming a single group of TSS (TCATCAT region) with a total expression value of 20.9 tags per million (tpm). We obtained similar results with the pro-SmAMP2*:uidA* sample, with 161 reads (0.001% of total number of reads) uniquely mapped to the pro-SmAMP2 promoter and also forming a single group of TSS (TCATCAT region) with a total expression value of 15.9 tpm. In general, we observed significant reproducibility between the two samples in TSS structure. Compared to our previous analysis of the TSS positions, we experimentally determined the TSS position in aseptic transgenic tobacco plants as being 4 bp closer to the TATA box than earlier thought [28].

We did not observe any alternative matching regions for the corresponding reads in the tobacco genome (GCF_000715135.1) validating the de novo expression of *uidA* under the control of the pro-SmAMP1 and pro-SmAMP2 promoters.

### 3.2. Comparative in silico Analysis of pro-SmAMP1 and pro-SmAMP2 Promoter Sequences

**Figure 2:**
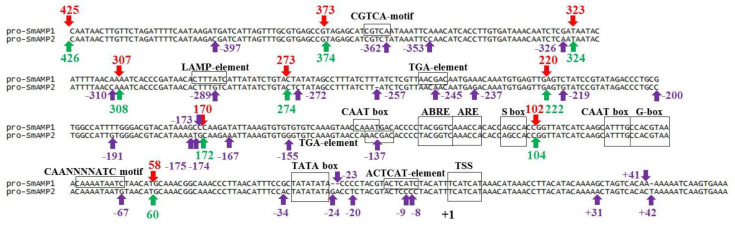
Alignment of pro-SmAMP1 and pro-SmAMP2 promoters and 5′-untranslated regions (UTRs). Letters in black above boxes indicate *cis*-acting elements. Purple arrows indicate point mutations; numbers above and below the arrows indicate their positions from the transcription start site (TSS) of the pro-SmAMP2 promoter. Red and green arrows show new deletion variants of the promoters; +1 is the first nucleotide of the TSS.

An alignment of pro-SmAMP1 and pro-SmAMP2 promoter sequences revealed that the core, proximal, and distal regions of both promoters (up to –425 and –426 bp from TSS, respectively) shared 94% identity and differed mainly by point substitutions, small insertions, and deletions (Figure 2).

Polymorphisms between the two promoters consisted of 29 nucleotides that fell outside of the canonical *cis*-element (TATA box), the core region, and the TSS, as well as a number of *cis*-elements of proximal region up to −137 bp from the TSS, including the CAAT box, G box, S box, anaerobic responsive element (ARE), and abscisic acid (ABA)-responsive element (ABRE).

The pro-SmAMP1 promoter nevertheless showed several substantial structural differences relative to the pro-SmAMP2 promoter. In the core region just upstream of the TATA box at position −34 bp, we identified an A-to-G substitution that might result in reduced light-mediated effectiveness of the prototype 13 bp TATA box sequence (TCACTATATATAG) based on transient expression in plants [37]. The substitution of a CC dinucleotide with an AT at the −9 and −8 bp positions may affect the ACTCAT *cis*-element, which normally induces gene expression in response to hypo-osmolarity and feeding with the amino acid proline [30]. The TATA box and TSS are located closer to each other in the pro-SmAMP1 promoter due to the deletion of two bp at positions –24 and –23. In the proximal region, a G-to-C substitution at −67 bp may generate a full CAANNNNATC motif in the pro-SmAMP1 promoter that might confer repression of promoter activity in dim light [38]. A C-to-T substitution at position −137 bp introduces a CAAT box in the pro-SmAMP1 promoter, whereas the pro-SmAMP2 promoter presents a TGA-element at the same location. A G-to-A substitution at −289 bp introduces a LAMP-element (also known as the antisense GATA element) in the pro-SmAMP1 promoter. Although both promoters contain a TGA-element, their location varies due to local polymorphisms: −245 bp for pro-SmAMP1 and −137 bp for pro-SmAMP2.

The nucleotide sequences of the *SmAMP1* and *SmAMP2* 5′-UTRs (56 and 57 bp-long, respectively), also differed by two substitutions and one deletion (Figure 2).

### 3.3. Identification of Smallest pro-SmAMP1 and pro-SmAMP2 Promoters for Effective Gene Expression in Agrobacterium-Mediated Plant Infiltration

**Figure 3:**
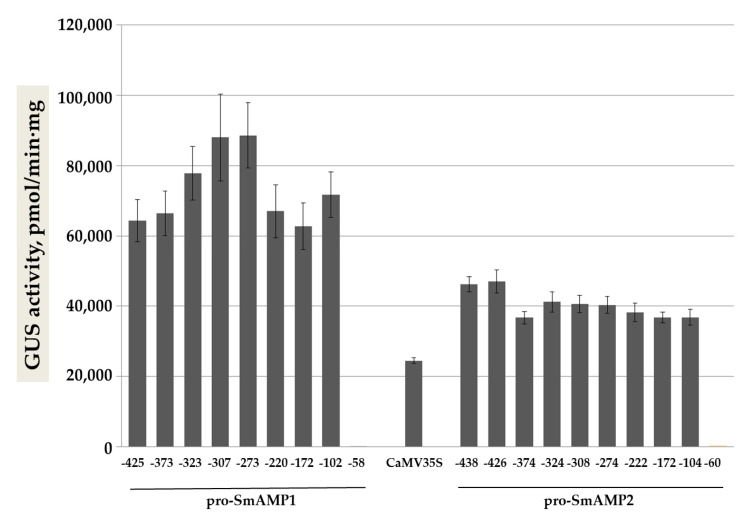
Activity of the GUS reporter in *N. benthamiana* leaves transiently expressing various deletion variants of the pro-SmAMP1 and pro-SmAMP2 promoters, alongside the CaMV35S viral promoter. Numbers indicate the length of the variants (in bp) from the TSS. Vertical lines show standard error, *n* = 44–60 for each construct. The plants were cultivated in a 16-h light/8-h dark photoperiod. Deletion variants pro-SmAMP1 (−425 bp) and pro-SmAMP2 (−438 bp) were obtained in course of previous studies [16,17].

To elucidate the functional organization of these promoters, we carried out a deletion analysis by truncating sequences from the 5’-end (Figure 2). We prepared deletion fragments of the pro-SmAMP1 (−58, −102, −170, −220, −273, −307, −323, and −373 bp) and pro-SmAMP2 (−60, −104, −172, −222, −274, −308, −324, −374, and −426 bp) promoters for cloning into the pCAMBIA1381z plasmid carrying the *uidA* reporter gene. We introduced the resulting constructs into the leaves of *N. benthamiana* plants by *Agrobacterium* (*Agrobacterium tumefaciens*)-mediated infiltration (Figure 3). 

We discovered that the shortest variants for both promoters (−58 and −60 bp from TSS) showed little expression (GUS activity was ca. 1.5 times higher than background 140–253 pmol/mg·min). The −102 and −104 bp promoter variants with high promoter activity were thus corresponding to smallest functional promoters and were designated as pro-SmAMP1 (102) and pro-SmAMP2 (104), respectively. Furthermore, we determined that shortening the pro-SmAMP1 promoter from −425 to −102 bp did not significantly affect the expression levels of the reporter gene. The pro-SmAMP1 (102) promoter variant exhibited GUS activity of 71,600 ± 5700 pmol/mg·min (Figure 3). Similarly, Figure 3 shows that truncating the pro-SmAMP2 promoter from −426 to −104 bp did not lower its effectiveness significantly, with a specific GUS activity measured for the pro-SmAMP2 (104) promoter variant of 36,800 ± 2200 pmol/mg·min. Across all promoter variants, the pro-SmAMP1 promoter was stronger than the pro-SmAMP2 promoter, as observed earlier [19].

### 3.4. New Deletion Variants of Pro-SmAMP1 and Pro-SmAMP2 Promoters Differ in the Expression of Selectable Markers in Transgenic Plant Cells on Selection Medium with Kanamycin

The *nptII* gene confers resistance to the antibiotic kanamycin and was used to test the usefulness of the new pro-SmAMP1 and pro-SmAMP2 promoter deletion variants in the selection of transgenic events. We cloned the promoter variants into the binary vector pCAMBIA2300, thus placing *nptII* expression under the control of the new pro-SmAMP1 and pro-SmAMP2 promoter deletion variants. We then introduced the vectors in *Agrobacterium* before infecting tobacco leaf explants, followed by selection on growth medium containing 350 mg/L kanamycin to score the regeneration of transgenic shoots (Table 1).

As indicated by results presented in Table 1, shortening the pro-SmAMP1 promoter did not change the effectiveness of *nptII*-mediated selection appreciably, with the exception of the deletion variant –102 bp, which led to an average of ca. 1.5 rooted shoots per explant after 3 months of cultivation. 

All deletion variants of the pro-SmAMP2 promoter, excluding −222 bp, resulted in more transgenic shoots than pro-SmAMP1 variants of similar length. Variants −426, −374, −172, and −104 bp showed the strongest differences relative to their matched pro-SmAMP1 length brethren. Surprisingly, the −426 bp variant was over twice as effective in transgenic shoot production compared to all other pro-SmAMP2 promoter variants. The pro-SmAMP2 (104) promoter, as defined earlier, regenerated ca. 4.7 shoots on average that were resistant to the antibiotic per explant. Consistent with our previous work, all pro-SmAMP2 promoter variants performed 2–3 fold better than pro-SmAMP1 variants for the selection of transformed tobacco cells, calli, and shoots in the presence of excess kanamycin.

**Figure 4:**
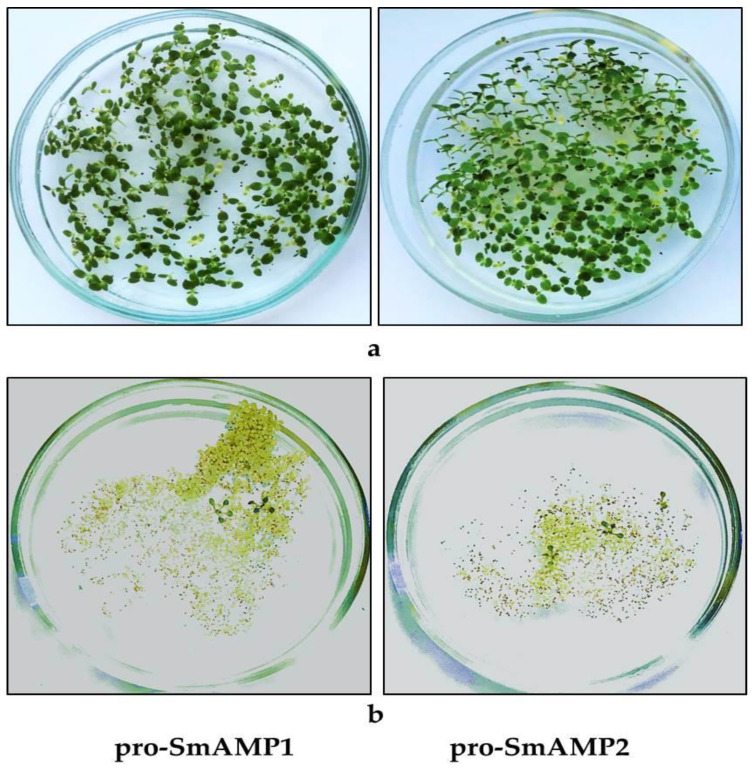
Segregation of T_1_ seedlings on selective medium containing kanamycin. (**a**) Progeny of primary tobacco transformants expressing *nptII* from the pro-SmAMP1 (102) and pro-SmAMP2 (104) promoters. (**b**) Primary *Arabidopsis* transformants after *Agrobacterium*-mediated transformation with pro-SmAMP1*:nptII* and pro-SmAMP2*:nptII* promoter variants, −170 and −172 bp from the TSS, respectively.

In order to determine the efficiency of the new promoter variants for the selection of tobacco transgenic seedlings, we collected seeds resulting from self-pollination of primary transformants. We observed clear segregation of kanamycin resistance among seedlings when T_1_ seeds were sown on selective medium (Figure 4a). In many cases, the segregation ratio was to 3:1, characteristic for the segregation of a T-DNA inserted at a single locus (Table A2 (Appendix B)).

We also transformed *Arabidopsis* plants with the same constructs. At the recommended kanamycin concentration of 50 mg/L [29], all constructs except those with –102 and –104 bp promoters provided effective selection of viable primary transformant seedlings within 7–10 days (Figure 4b). In the case of the pro-SmAMP1 (102) and pro-SmAMP2 (104) promoters, several green seedlings appeared that were bigger than kanamycin-sensitive seedlings; however, they later bleached and failed to grow in soil. In the next generation, T_2_ seedlings carrying deletion variants of both promoters with lengths from –426 to –170 bp segregated into kanamycin-resistant and -sensitive, in many cases in a 3:1 ratio indicative of monogenic inheritance (Table A3).

All kanamycin resistant tobacco (T_1_-progeny) and *Arabidopsis* (T_2_-progeny) plants (17–20 pieces for each variant construct) were free from *Agrobacterium* contamination and, according to PCR data (data not shown), all contained the hybrid nucleotide sequences between the corresponding promoter variants and *nptII*. 

### 3.5. Identification of Mutations Causing High-Level Transient Expression of the Reporter Gene

**Figure 5:**
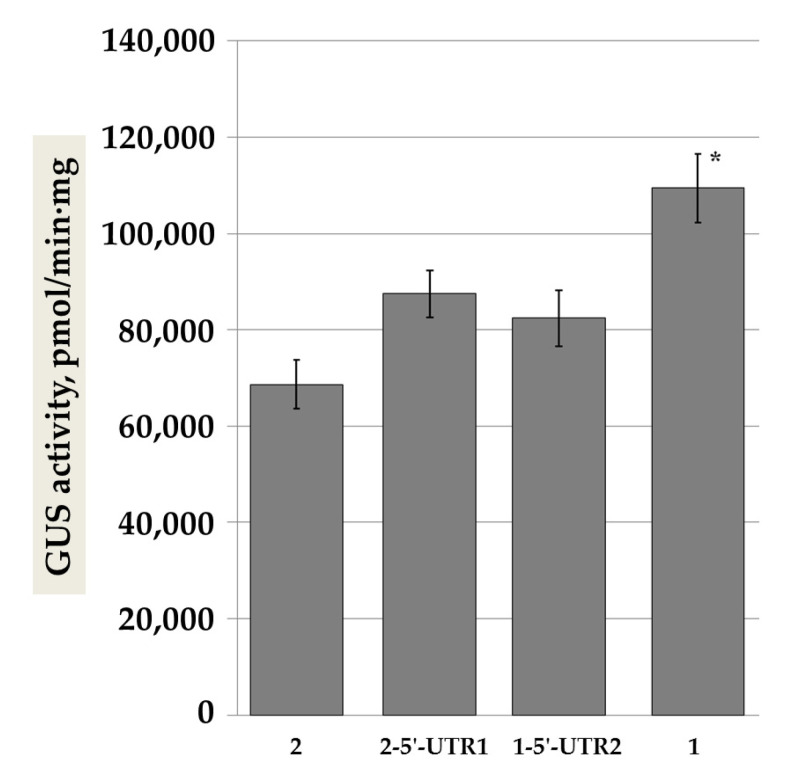
Activity of the GUS reporter in *N. benthamiana* leaves transiently expressing the pro-SmAMP1 (102) and pro-SmAMP2 (104) promoters and the 1-5′-UTR1 and 2-5′-UTR2 chimeric variants. The intact pro-SmAMP1 (102) and pro-SmAMP2 (104) promoters are indicated by the numbers 1 and 2, respectively. Vertical lines show standard errors, *n* = 80 for each construct. The plants were cultivated in a 16-h light/8-h dark photoperiod. The asterisk marks significant difference relative to pro-SmAMP2 by Student’s *t*-test (* *p* = 0.05).

To evaluate the influence of nucleotide polymorphisms on gene expression levels, we decided to separately study polymorphisms from the promoter regions and from the 5′-UTRs. We thus generated two chimeric constructs. The first one consisted of the pro-SmAMP1 (−102 bp) promoter region and the 57 bp 5′-UTR from the *SmAMP2* and was named 1-5′-UTR2. The second chimeric construct contained the pro-SmAMP2 (–104 bp) promoter region and the 56 bp 5′-UTR from *SmAMP1* and was named 2-5′-UTR1. Both chimeric constructs drove expression of the *uidA* reporter to the same level as the pro-SmAMP2 promoter of the same length in transient assays in *N. benthamiana* leaves, although the pro-SmAMP1 promoter resulted in higher *uidA* expression, and thus GUS activity (Figure 5).

The data presented in Figure 5 suggest that the 5′-UTR from *SmAMP1* has a positive effect on the expression level or mRNA stability of the *uidA* reporter or on the accumulation of the GUS protein in plant cells. However, this can only partially account for the higher performance of pro-SmAMP1 over pro-SmAMP2. 

**Figure 6:**
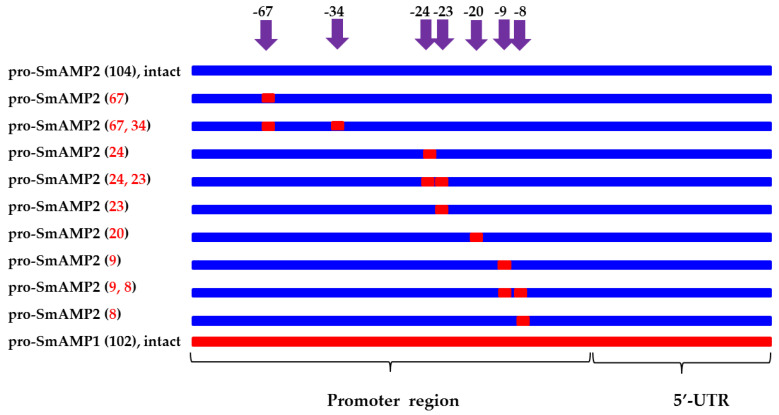
Design of new synthetic variants of the pro-SmAMP2 promoter (blue) with point substitutions or deletions of nucleotides characteristic of the pro-SmAMP1 promoter (red). The 5′-UTR is the 5′-untranslated region of *SmAMP1* or *SmAMP2* genes. Purple arrows indicate point mutations; numbers near the arrows indicate their position from the pro-SmAMP2 promoter TSS.

To functionally characterize the polymorphisms in the promoter regions by directed mutagenesis, we generated nine new synthetic variants of the pro-SmAMP2 promoter (together with the *SmAMP2* 5′ UTR) that introduced the nucleotides seen in the pro-SmAMP1 promoter (see Figure 2 and Figure 6).

**Figure 7:**
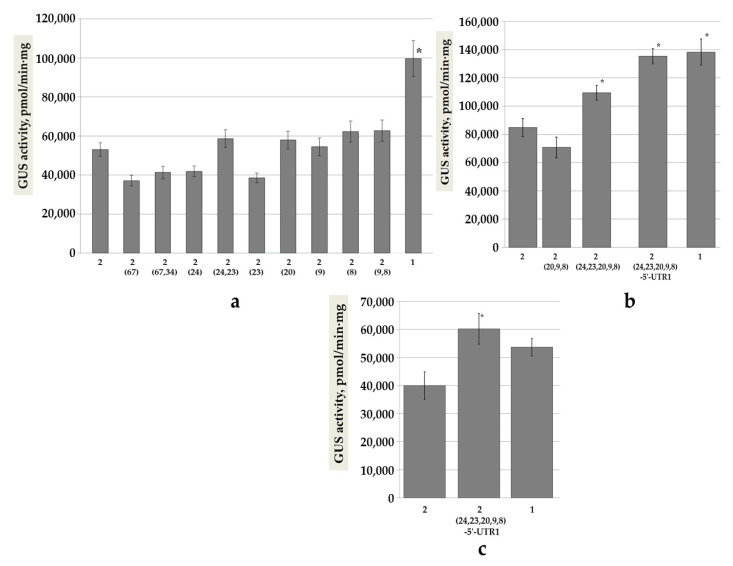
GUS activity from the *uidA* reporter expressed in *N. benthamiana* leaves in transient assays. The intact pro-SmAMP1 (102) and pro-SmAMP2 (104) promoters are indicated by the numbers 1 and 2, respectively. The variants created on the basis of pro-SmAMP2 (104) are designated as number 2 with the position of the introduced mutation(s) in parentheses. (**a**) Synthetic promoter variants with single and double bp mutations. The plants were grown in a 16-h light/8-h dark photoperiod; (**b**) synthetic and chimeric variants with three or more mutations. The plants were grown in a 16-h light/8-h dark photoperiod; (**c**) synthetic chimeric variant of the pro-SmAMP2 promoter with five mutations and the 5′-UTR from *SmAMP1*. The plants were grown in an 8-h light/16-h dark photoperiod. Vertical lines show standard errors, *n* = 32–60 for each construct. The asterisks mark the values that significantly exceed those for pro-SmAMP2 by Student’s *t*-test (* *p* = 0.05).

We named the new constructs according to the nucleotide position of the single (−67; −24; −23; −20; −9; −8) or double (−67 and −34; −24 and −23; −9 and −8) mutations introduced. Their comparative evaluation by transient expression in *N. benthamiana* leaves revealed that all new constructs were inferior to the intact pro-SmAMP1 promoter (Figure 7a).

The deletions of single nucleotides at the −24 or −23 positions reduced the effectiveness of the pro-SmAMP2 promoter, but the simultaneous deletion of both nucleotides −24 and −23 bp (as in the intact pro-SmAMP1 promoter) returned the promoter effectiveness to that of the intact pro-SmAMP2 promoter (Figure 7a). The substitutions of single nucleotides at positions −20, −9, or −8 or substitutions at both −9 and −8 positions did not significantly affect promoter efficiency. Although the ACTCAT *cis*-element was introduced into the pro-SmAMP2 (9,8) synthetic variant, this addition proved insufficient to enhance promoter efficiency. The introduction of the CAANNNNATC motif by substitutions at positions –67 and/or –34 reduced the promoter efficiency by 29%, although this difference was not statistically significant.

These results suggested that the higher performance of the pro-SmAMP1 promoter during transient expression assays was probably caused by the ACTCAT *cis*-element located between the TATA box and the TSS, requiring a specific nine-nucleotide 5′-context for proper function [30]. However, a substitution at position −20 did not affect the activity of the pro-SmAMP2 promoter (Figure 7a). We next hypothesized that the higher performance of the pro-SmAMP1 promoter may be a consequence of the position of the ACTCAT *cis*-element relative to the TATA box. The distance between the ACTCAT and TATA box sequences can be changed from pro-SmAMP2-type to pro-SmAMP1-type by the deletion of nucleotides −24 and −23 (Figure 2). To test this hypothesis, we generated two new variants of the pro-SmAMP2 promoter: the first was pro-SmAMP2 (20,9,8) with substitutions in positions −20, −9, and −8, while the second variant was pro-SmAMP2 (24,23,20,9,8), with an additional two deletions at positions −24 and −23 bp. We discovered that the combination of the three substitutions at positions −20, −9 and −8 did not affect the effectiveness of the pro-SmAMP2 promoter (Figure 7b). However, shortening the distance between ACTCAT *cis*-element and the TATA box by the additional deletion of positions –23 and –24 in the pro-SmAMP2 (24,23,20,9,8) variant significantly raised promoter output relative to the intact pro-SmAMP2 promoter, although not to the same level as the pro-SmAMP1 promoter. Last, we combined the pro-SmAMP2 (24,23,20,9,8) variant with the *SmAMP1* 5′-UTR, named pro-SmAMP2 (24,23,20,9,8)-5′-UTR1. This variant matched the intact pro-SmAMP1 promoter output, as evidenced by measured GUS activity, although it contained neither the CAANNNNATC motif (the substitution at –67 bp) nor the substitution at –34 bp known as potential repressors of gene expression in shorter photoperiods [37,38].

To evaluate the influence of the CAANNNNATC motif and of the substitution at −34 bp in shorter photoperiods (8/16 hours), we compared the efficiencies of the pro-SmAMP1, pro-SmAMP2, and pro-SmAMP2 (24,23,20,9,8)-5′-UTR1 constructs in transient expression assays in *N. benthamiana* leaves. Shortening day length led to a reduction in GUS activity for all variants (Figure 7c). The pro-SmAMP1 promoter was not statistically different from the pro-SmAMP2 promoter. Notably, the pro-SmAMP2 (24,23,20,9,8)-5′-UTR1 variant performed better than pro-SmAMP2 and comparable to the pro-SmAMP1 promoter.

### 3.6. Identification of Mutations Determining Constitutive Expression of the Selectable Marker Gene in Transgenic Plant Cells on Medium with Kanamycin

To determine which features of promoter architecture controlled the constitutive expression of the pro-SmAMP2 promoter and its superiority for the selection of transgenic individuals, we used the *nptII* selectable marker from the pCAMBIA2300 vector under the control of the synthetic variants of the pro-SmAMP2 promoter, containing point mutations (67), (23), and (24) alone or in combination, (67,34), (24,23), (20,9,8), and (24,23,20,9,8), as well as the chimeric variant with combination (24,23,20,9,8)-5′-UTR1.

**Figure 8:**
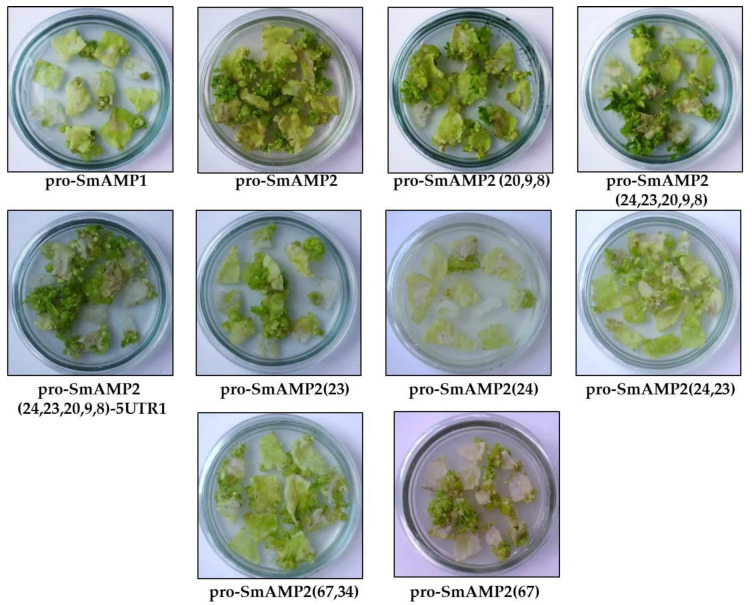
Efficiency of selection of tobacco calli using the intact pro-SmAMP1 (102) and pro-SmAMP2 (104) and synthetic promoters on selective growth medium containing 550 mg/L kanamycin. Synthetic and chimeric variants based on the pro-SmAMP2 (104) promoter are indicated by the nucleotide position of the respective mutations in parentheses. The explants were incubated in a 16-h light/8-h dark photoperiod and 22–24 °C. *n* = 40 for each construct.

To accelerate analysis, we increased the kanamycin concentration in the growth medium to 550 mg/L, allowing an easy visual differentiation of the promoter activities based on the efficiency of callus formation and their morphology, thus avoiding the tedious process of tissue culture and production of transgenic shoots (Figure 8).

In transformed tobacco explants cultivated on selective medium for two months, numerous green calli and morphogenetic structures formed with the intact pro-SmAMP2 (104) promoter and its derivatives with mutations (23), (24,23), (67), (67,34) (20,9,8), (24,23,20,9,8), and (24,23,20,9,8)-5′-UTR1. In this group of promoters, the variants (23) and (24,23) were less effective than the others. We observed that the intact pro-SmAMP1 (102) promoter and the synthetic pro-SmAMP2 (24) variant were not capable of producing transgenic shoots. The deletions of the nucleotides at either the −24 or −23 positions reduced *nptII* expression in the context of the pro-SmAMP2 promoter variant. However, these single deletions do not reflect the sequence of the intact pro-SmAMP1 promoter. The simultaneous deletion of the nucleotides at positions −24 and −23 recovered constitutive expression of the pro-SmAMP2 (24,23) synthetic variant. Unlike the intact pro-SmAMP1 promoter, which also carries these two deletions, the pro-SmAMP2 (24,23) synthetic variant allowed the development of morphogenetic calli.

Nucleotide substitutions at –67 (thus inserting a CAANNNNATC motif) and at −34 had detrimental but non-critical effects on the ability of the pro-SmAMP2 (67) and (67,34) synthetic variants to drive *nptII* expression during the selection of transformed tissues.

Surprisingly, the variants pro-SmAMP2 (24,23,20,9,8) and pro-SmAMP2 (24,23,20,9,8)-5′-UTR1, which contain the active ACTCAT *cis*-element, did not reach higher effectiveness for selection of transformants, although they lack potential repressors such as the CAANNNNATC motif and the substitution at the −34 position and had out-performed the pro-SmAMP2 promoter in transient expression assays. The synthetic variant pro-SmAMP2 (20,9,8) also contained the ACTCAT *cis*-element (though not functional in transient expression assays, see Figure 7b) and also exhibited reduced performance.

### 3.7. Functional Validation of the Role of the ACTCAT Cis-Element in the pro-SmAMP1 Promoter under Proline Stress Conditions

**Figure 9:**
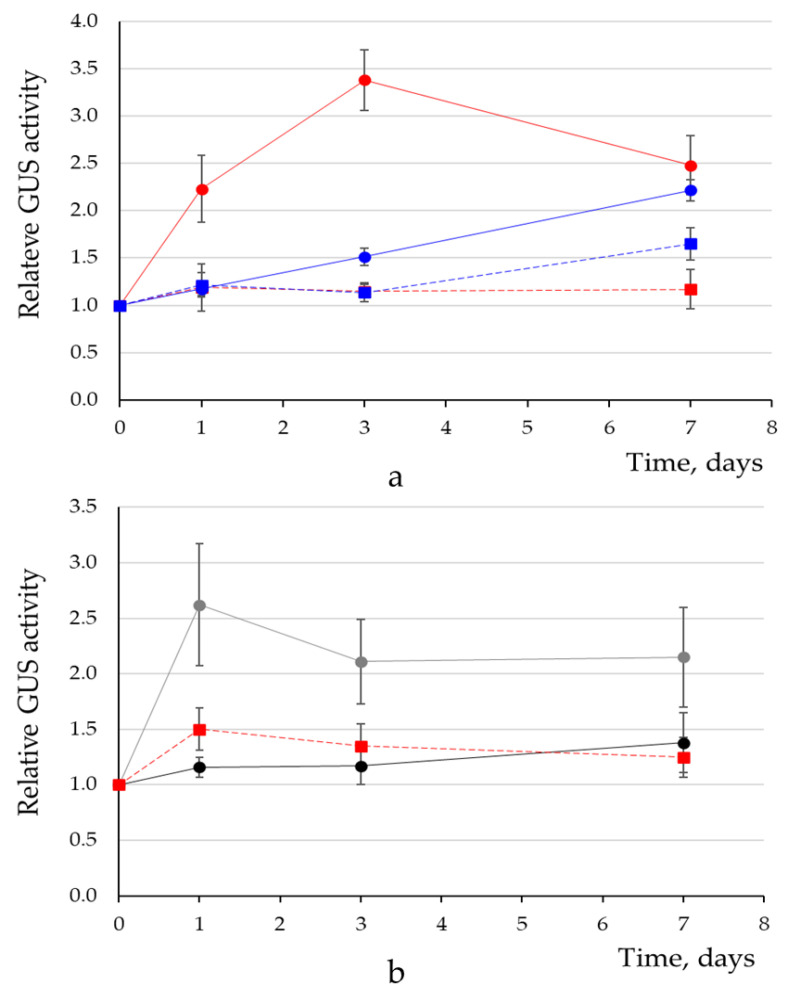
Relative GUS activity levels over time in the leaves of transgenic tobacco plants grown aseptically upon addition of 90 mM proline or sterile water (control). (**a**) Treatment with a freshly prepared proline solution of plants carrying the pro-SmAMP1*:uidA* (proline—red circles, *n* = 16, control—red squares, *n* = 16) and the pro-SmAMP2*:uidA* construct (proline—blue circles, *n* = 22, control—blue squares, *n* = 22). (**b**) Treatment of plants bearing the pro-SmAMP1:*uidA* construct by proline solution that had been stored for 5 months (gray circles, *n* = 12), or 10 months (black circles, *n* = 12), or by sterile water (red squares, *n* = 24). The plants were grown in a 16-h light/8-h dark photoperiod. GUS activity in leaves before treatment was set to 1.

To examine the function of the ACTCAT *cis*-element in the core promoter, we produced transgenic tobacco T_1_ plants carrying a single T-DNA copy expressing the *uidA* reporter gene under the control of the pro-SmAMP1 (102) or pro-SmAMP2 (104) promoters. We observed a mild increase (1.3 ± 0.1 times) in GUS activity from detached leaves of pro-SmAMP1*:uidA* transgenic tobacco plants that had been exposed to 90 mM proline for 24 hours in liquid medium under dim light conditions. We performed the same experiment under stronger light conditions (150 μmol/m^2^), resulting in a 1.6-fold increase (±0.3) with the pro-SmAMP1 promoter and a 1.3-fold increase (±0.1) with the pro-SmAMP2 promoter. We hypothesized that the activation of the promoters required strong light for over 24 hours. We therefore repeated the experiment with transgenic tobacco plants grown on MS solid medium and with enough leaves to analyze reporter activity after one, three, and seven days after proline treatment. We discovered that the promoters conferred different expression patterns to the reporter gene after addition of a freshly-prepared proline solution (Figure 9a).

After the addition of proline, we noticed that GUS activity in the leaves of pro-SmAMP1*:uidA* transgenic plants increased during the first three days, with a maximum normalized activity of 3.4 times that measured at time 0. However, GUS activity subsequently decreased after seven days to 2.5 times the activity levels seen at time 0. In the control group (treated with sterile water instead of proline), we only observed a small increase in GUS activity after the first day. GUS activity in proline-treated plants was significantly different from GUS activity measured for the control group between day 1 and day 7 (*p* = 0.05).

We observed a linear increase for GUS activity in the leaves of pro-SmAMP2*:uidA* transgenic plants after proline addition over the course of the experiment. After seven days, GUS activity was 2.2-fold higher than at time 0. The addition of sterile water to the medium did not affect GUS activity in the leaves of control transgenic plants. 

We hypothesized that the rapid decrease of pro-SmAMP1 promoter activity after seven days might result from the accumulation of proline oxidation products in plant cells. To test this hypothesis, we treated pro-SmAMP1*:uidA* transgenic tobacco plants with a proline solution that had been stored at + 4 °C for 5 or 10 months (Figure 9b), which was accompanied by a lower overall increase in GUS activity, presumably due to the spontaneous oxidation products from proline. Employing a proline solution stored for 10 months completely suppressed the activation of pro-SmAMP1*:uidA* expression, as evidenced by the low GUS activity in both water control and proline-treated plants.

## 4. Discussion and Conclusions

As the shortest versions of the pro-SmAMP1 and pro-SmAMP2 promoters (the −58 and −60 bp fragments, respectively) proved to be weak, we selected the longer fragments of –102 and –104 bp as smallest promoter units, since they largely preserved the characteristics of the original promoters, for routine use in plant biotechnology (Figure 2). The CAAT box and/or G-box *cis*-elements present in these promoter fragments from −104 to −58 bp and 50–60 bp upstream of the TATA box are critical for the efficient operation of both promoters. This result should not be surprising, as the G-box, depending on nucleotide context, confers high-level constitutive expression to promoters, while its absence turns the CaMV35S (−90 to +8 bp) promoter into a weak promoter [39]. We confirm that genome editing of G-box elements or their flanking nucleotide sequences will enable targeted changes to gene expression [26]. The CAAT box *cis*-element is necessary to stabilize transcription complexes, and its absence 40–50 bp upstream of the TATA box reduces the efficiency of the nos promoter by a factor of about 20 [40,41]. In the pro-SmAMP1 and pro-SmAMP2 promoters, these two *cis*-elements are adjacent, and their target transcription factors may prevent their respective binding because of steric hindrance [27,42]. We therefore cannot rule out the possibility that only one element is truly functional in the operation of these two promoters.

The barrelclover (*Medicago truncatula*) MtHP promoter, consisting of only 107 bp, was previously shown to support the expression of the reporter gene with an efficiency about half that of the CaMV35S promoter, but its suitability for expression of selectable marker genes has not yet been reported [13]. Our results clearly suggest that the pro-SmAMP1 and pro-SmAMP2 deletion variants −102 and −104 bp, respectively, are worth considering for the target gene expression, as their efficiency was 50% higher than that of CaMV35S (Figure 3). The same variants are also suitable for the selection of tobacco transgenic events on growth medium containing 550 mg/L kanamycin, although with various levels of efficiency (Figure 8). In earlier research, 350 mg/L kanamycin was reported to be excessive for selecting tobacco transformants, but made it possible to reliably distinguish between pro-SmAMP1 and pro-SmAMP2 promoters based on the number of regenerating transgenic shoots [19]. In the present study, a concentration of 550 mg/L kanamycin allowed the visual differentiation of the transgenic calli resulting from transformation with the pro-SmAMP1 and pro-SmAMP2 promoters, thus reducing the time required for analysis by several months.

We determined that the proximal sequences of the pro-SmAMP1 and pro-SmAMP2 promoters may play an important role in the expression of the selectable marker gene at a more constitutive level. For instance, the sequence from −172 to −102 bp in both promoters are important for the constitutive expression of the *nptII* gene at the seedling stage in *Arabidopsis* (Figure 4b) and contains a number of well-known *cis*-elements: S box, ARE, and ABRE (Figure 2). In the future, the consecutive, stepwise inactivation of these *cis*-elements by site-directed mutagenesis or investigation of the chimeric promoters (having the 35S minimal promoter inside them as a core promoter) will allow a thorough dissection of their role in promoter efficiency for selecting transgenic plant cells at that developmental stage.

The proximal region of the pro-SmAMP2 promoter, from –426 to –374 bp, acts as a powerful positive element for tobacco selection on kanamycin-containing medium when the sequence from −438 to −426 bp is missing (Table 1), but this did not hold true for *Agrobacterium*-mediated leaf infiltration. Since we did not witness a similar pattern for the pro-SmAMP1 promoter, the high constitutive expression level of the selectable marker gene does not rule out a hierarchy of *cis*-elements from different regions of the pro-SmAMP2 promoter and may be the result of a specific interaction between several such elements in calli. According to the PLACE database of promoter elements [35], the substitution of one nucleotide at the −397 position does not introduce or interrupt known *cis*-elements in the pro-SmAMP1 or pro-SmAMP2 promoters. However, one cannot rule out the possibility that a new, unknown *cis*-element might have been inserted or destroyed as a result of this mutation.

The structure of the 5′-UTRs mRNA is essential for the accumulation of recombinant proteins in plant cells. For instance, the 5′-UTR of the l-aminocyclopropane-l-carboxylate synthase (*ACS1*) gene from mung bean (*Vigna radiata*) enhances the translation of the reporter protein in tobacco, *Arabidopsis*, and *V. radiata* [43]. It is comparable in terms of efficiency with the 5′-UTR of the *chlorophyll a/b binding protein* (*Cab22L*) gene from *Petunia x hybrida* (Mitchell) (a well-known translation enhancer) and is five times more effective than the 5′-UTR of the *PECTIN ACETYLESTERASE* (*PAE*) gene from *V. radiata* [18,43]. In the present work, the three polymorphisms that distinguish the 5′-UTR of *SmAMP1* from the 5′-UTR of *SmAMP2* are functional, and it is likely that they determine the formation of the RNA secondary structure to influence the efficiency of ribosome binding and recombinant protein translation [43]. These results are consistent with our previous data on the positive dependence of GUS activity on the accumulation of *uidA* mRNA in homozygous transgenic tobacco lines [16,17]. This functional dependence exhibited comparable and strong correlation ratios of *r* = 0.8–0.9 for both promoters, but the regression ratio was higher for pro-SmAMP1 (*b* = 5.5) than for pro-SmAMP2 (*b* = 2.4). Thus, transgenic plants grown in a greenhouse accumulated a greater amount (approx. 22%) of the reporter protein when driven by the pro-SmAMP1 promoter [19]. It is worth noting that a close link between mRNA levels and the levels of the encoded protein product cannot always be drawn in eukaryotic cells [44].

During earlier in silico analysis, the ACTCAT *cis*-element was identified in the promoter regions of many genes that were induced during rehydration and hypo-osmolarity stress [45,46]. However, the positive activity of this element in response to the effect of exogenous proline or hypo-osmolarity was only confirmed by experiment in the distal and proximal regions of the *Arabidopsis PROLINE DEHYDROGENASE* (ProDH) promoter [30] and in the ζ-carotene desaturase (ZDS) promoter in the green alga *Dunaliella bardawil* [46]. The present work is the first to demonstrate that the ACTCAT *cis*-element is functional in the core region of the pro-SmAMP1 promoter and has a pleiotropic effect, making this promoter statistically more efficient in *Agrobacterium*-mediated infiltration of *N. benthamiana* leaves (Figure 2 and Figure 7). The pro-SmAMP1 promoter also changes the expression profile of the *uidA* gene in transgenic tobacco plants when exogenous proline is added (Figure 9) and has an adverse effect on the constitutive expression of the *nptII* gene during the selection of transgenic events (Figure 8).

The potential of the ACTCAT *cis*-element to activate the ProDH promoter when exposed to proline has previously been shown to depend on the flanking 5’-nucleotide context within a 9-bp window [30]. ACTCAT *cis*-elements from two distal sites of the ProDH promoter with different flanking contexts were not fully identical in terms of their functionality. The findings of another study involving maize (*Zea mays* L.) showed that there were differences in the ability of the putative transcription factor ZmbZIP91 to bind to the ACTCAT *cis*-element within the distal and proximal regions of the starch synthase I (*SSI*) promoter [47]. The latter observation suggests that the sequence flanking the ACTCAT *cis*-element influences transcription factor binding affinity. In the present work, the 9 bp-long 5′-nucleotide context of the ACTCAT *cis*-element differed within the pro-SmAMP1 promoter core from a similar site in the pro-SmAMP2 promoter by one nucleotide substitution at the −20 position (Figure 2). However, this substitution alone was not sufficient to enhance the efficiency of the synthetic variant of the pro-SmAMP2 (20,9,8) promoter (Figure 7b).

The activation of gene expression by the ACTCAT *cis*-element is known to increase or decrease as the distance between the distal region and the TATA box of the ProDH core promoter diminishes in transgenic tobacco and *Arabidopsis* plants [30]. The ACTCAT *cis*-element has almost no effect if located in a proximal position (~62 bp) to the TATA box. Our results suggest that in order to function as expected, the ACTCAT *cis*-element should be precisely positioned in the core promoter, in particular, in relation to the TATA box. Since the ACTCAT *cis*-element is located in the core of the pro-SmAMP1 promoter, it is impossible to exclude the possibility that its binding by transcription factors may play a role in the assembly of the preinitiation complex (PIC) [48]. Various PICs differing in protein composition can initiate transcription with different efficiencies and can likely start from different TSS sequences [49], which does not contradict the CAGE results (Figure 1). It is possible that the protein composition of the PIC at the pro-SmAMP1 promoter changes upon treatment with proline, which determines its varying efficiency. This hypothesis requires experimental confirmation by comparing the protein composition of the PIC and the quantitative characterization of the TSS of the pro-SmAMP1 promoter before and within several days after the addition of exogenous proline. 

In an earlier study, the reporter activity was shown to increase from 3 to 45 times in one day in transgenic plants when exposed to 90 mM exogenous proline if using a ProDH promoter variant with two ACTCAT *cis*-elements in its distal region [30]. Moreover, the methods provided stated that seedlings or detached tobacco leaves were incubated in MS liquid medium without sucrose and in dim light [50]. In the present work, we recorded a significant increase in GUS activity (up to 3.4-fold) during the first three days in intact leaves of transgenic tobacco plants expressing pro-SmAMP1:*uidA*, but our plants were grown in MS medium with sucrose and in bright light (Figure 9a). Thus, the ProDH and pro-SmAMP1 promoters share the same positive response to exogenous proline, although the intensity and conditions of their responses differ. The need for bright light in the activation of the pro-SmAMP1 promoter may be explained by the presence in its sequence of the CAANNNNATC motif, which has a repressive effect [38], and which may limit the efficiency of the promoter in dim light and when the photoperiod is shorter (Figure 7c).

In the present study, GUS activity from tobacco leaves expressing pro-SmAMP1:*uidA* followed a biphasic profile in response to proline treatment, first increasing and later decreasing (Figure 9b). Proline is known to become oxidized to the intermediate compound, Δ′-pyrroline-5-carboxylate (P5C), in plant cell mitochondria by proline dehydrogenase [51]. This oxidation explains the key toxic effect of proline: *Arabidopsis* Pat(B33)-*Gus* transgenic plants and *reduced sugar response1-1* (*rsr1-1*) mutants are known to be killed when less than 1 mM of P5C is added [52]. Thus, the lower efficiency of the pro-SmAMP1 promoter by day 7 may be the result of the accumulation of a critical concentration of proline oxidation products (Figure 9a). This hypothesis was verified by using an aged aqueous proline solution (Figure 9b). The importance of these results is that they explain why the pro-SmAMP1 promoter may be stronger for *Agrobacterium*-mediated infiltration but less constitutive than the pro-SmAMP2 promoter for the selection of transgenic events on selective kanamycin medium.

Free proline content increases by one or sometimes two orders of magnitude under drought or salt stress, or during exposure to low temperatures, heavy metals, herbicides, antibiotics, or pathogens (including *Agrobacterium*) [53,54,55,56]. Thus, we next hypothesized that the significantly higher performance of the pro-SmAMP1 promoter over pro-SmAMP2 for *Agrobacterium*-mediated infiltration of *N. benthamiana* may stem from the activation of the pro-SmAMP1 promoter via the ACTCAT *cis*-element in response to the accumulation of endogenous proline brought upon by *Agrobacterium* infection. Since the samples for the measurement of GUS activity in *Agrobacterium*-infiltrated plants were taken on day 7 (see Materials and Methods), it follows that P5C had not reached the critical levels required for significant inhibition of the pro-SmAMP1 promoter by that time.

During the transformation of tobacco cells by *Agrobacterium* infection with pro-SmAMP1:*nptII* and pro-SmAMP2:*nptII*, the two promoters encountered another set of conditions that affected the results (Table 1, Figure 8). After two days of co-cultivation of explants with *Agrobacterium*, we added the antibiotic timentin to eliminate *Agrobacterium* (see Materials and Methods), presumably removing a potential signal that would have caused the release of sufficiently high concentrations of free proline and then of the proline oxidation products to inhibit the pro-SmAMP1 promoter. However, selection of transformants with kanamycin is indisputably a stress factor for explants, which are mainly composed of non-transformed cells. As the selection lasts several months, non-transformed explant tissues are a likely source of endogenous proline and of proline oxidation products that will then repress the pro-SmAMP1 promoter. However, kanamycin probably does not act as a stressor on transgenic cells and, regardless of whether the selectable marker gene is driven by the pro-SmAMP1 or pro-SmAMP2 promoter, they probably do not accumulate significant quantities of proline and of proline oxidation products. Our observations showed that green calli generated with either promoter and detached from the non-transformed explant tissues on selection medium containing 550 mg/L kanamycin did not differ visually from each other after one month.

We previously reported that the expression of the *SmAMP1* and *SmAMP2* genes was high and increased when *S. media* plants were infected by pathogenic fungi [27]. Six days after inoculation with fungi, *SmAMP1* expression increased 10 to 70 times, whereas *SmAMP2* expression increased only 2–5 times. These results suggest that the accumulation of proline in response to pathogen infection in *S. media* may also explain the high inducibility of the pro-SmAMP1 promoter.

The CAANNNNATC motif determines the circadian pattern of several promoters of the *Lhc* gene family in tomato (*Solanum lycopersicum*) and acts as a negative regulator in dim light [38]. The A-to-G substitution in the prototypical 13 bp TATA-box sequence (TCACTATATATAG) was reported to have a similar effect in transient expression [37]. Our findings suggest that the CAANNNNATC motif and the substitution at the –34 position in the pro-SmAMP2 (67) and pro-SmAMP2 (67,34) promoter variants reduce the efficiency of both selection of transgenic events by kanamycin (Figure 8) and reporter expression during *Agrobacterium*-mediated infiltration (Figure 7a). 

In conclusion, when creating new promoters for routine use in genetic engineering, we propose that the CAANNNNATC motif and ACTCAT *cis*-element be excluded, and that negatively-acting sequences should be mutated from the promoter sequence if necessary to maximize promoter efficiency, especially under stress and short photoperiod conditions.

## Figures and Tables

**Table 1:** Efficiency of regeneration and selection of transgenic tobacco shoots (mean ± SD) with various deletion variants of the pro-SmAMP1 and pro-SmAMP2 promoters.

Genetic Construct (pCAMBIA2300)		Number of Shoots in Growth Medium with 350 mg/L Kanamycin		
		Total	Per Explant	
			Total	Rooted
Promoter	Deletion Variant, bp			
pro-SmAMP1	−425	248	5.5 ± 0.9	2.4 ± 0.6
	−373	194	4.3 ± 0.7	1.7 ± 0.4
	−323	266	5.9 ± 1.2	2.1 ± 0.6
	−307	180	4.0 ± 0.6	1.6 ± 0.4
	−273	249	5.5 ± 0.5	2.5 ± 0.4
	−220	293	6.5 ± 0.8	3.3 ± 0.5
	−170	239	5.3 ± 0.9	2.3 ± 0.6
	−102	81	1.8 ± 0.6	1.5 ± 0.3
pro-SmAMP2	−438	523	11.6 ± 1.3	6.2 ± 0.9
	−426	796	17.7 ± 1.7	13.5 ± 1.4 *
	−374	478	10.6 ± 1.2	6.8 ± 0.9 *
	−324	378	8.4 ± 1.0	3.8 ± 0.5
	−308	262	5.8 ± 1.5	3.7 ± 1.0
	−274	339	7.5 ± 1.0	3.8 ± 0.7
	−222	393	8.7 ± 0.9	3.9 ± 0.5
	−172	370	8.2 ± 0.8	5.2 ± 0.7 *
	−104	330	7.3 ± 0.9	4.7 ± 0.6 *

* Significant differences from the pro-SmAMP1 promoter of corresponding length (*p* = 0.05).

## Appendix A

**Table A1:** Primers for creation of new deletion, chimeric, and synthetic variant promoters.

Promoter Variant pro-SmAMP		Primer	
		Designation	Sequence in 5′–3′ Direction
**1**	425	The deletion variant was created earlier and was originally designated as 481 bp [16]	
	373	F375(amp1)	gaattctagagcatcgtcaataaa
		Rev(amp1)	ccatggtttcacttgatttttttg
	323	F325amp1	gaattccaatctcgataatacatttt
		Rev(amp1)	
	307	F309(amp2)	gaattcaaatcacccgataacact
		Rev(amp1)	
	273	F275amp1	gaattctatatagcctttatctttatctcg
		Rev(amp1)	
	220	F222amp1	gaattcagtctatccgtatagaccct
		Rev(amp1)	
	170	F172amp1	gaattccaagatattaaagtgtgtgt
		Rev(amp1)	
	102	F104(amp2)	gaattcggttatcatcaagcattt
		Rev(amp1)	
	58	F62amp2	gaattcgcaaacggcaaacc
		Rev(amp1)	
	5′-UTR2	A1	gaattcggttatcatcaagcatttgccacgtaaacaaaataatctaacatgcaaacggcaaaccctt
		B1	tttatgatgaaatgtagatgagtacgtaggggtatatatagcggaaatgttaagggtttgccgtt
		D51	
**2**	438	The deletion variant was created earlier and was originally designated as 455 bp [17]	
	426	F428(amp2)	gaattcataacttgttctagattttcaataag
		Rev(amp2)	ccatggtttcacttgatttttagt
	374	F376(amp2)	gaattctagagcatcgtctataaattcc
		Rev(amp2)	
	324	F326(amp2)	gaattctaatacattttaaccaaatcacc
		Rev(amp2)	
	308	F310(amp2)	gaattcaaatcacccgataacact
		Rev(amp2)	
	274	F276(amp2)	gaattctctatagcctttatcttatctcg
		Rev(amp2)	
	222	F224(amp2)	gaattcagtgtatccgtatag
		Rev(amp2)	
	172	F174(amp2)	gaattccaagaaattaaagtgtgg
		Rev(amp2)	
	104	F106(amp2)	gaattcggttatcatcaagcattt
		Rev(amp2)	
	60	F62(amp2)	gaattcgcaaacggcaaacc
		Rev(amp2)	
	5UTR1	A	gaattcggttatcatcaagcatttgccacgtaaacaaaataatgtaacatgcaaacggcaaaccctt
		F	tttatgatgaaatgtaggggagtacgtagaggtctatatatagtggaaatgttaagggtttgccgttt
		U1	ccatggtttcacttgatttttttgtgactagcttttgtatgtaaggtttatgtttatgatgaaatgt
	(67)	69	gaattcggttatcatcaagcatttgccacgtaaacaaaataatc
		Rev(amp2)	
	(67,34)	A36	gaattcggttatcatcaagcatttgccacgtaaacaaaataatctaacatgcaaacggcaaaccctt
		C36	tttatgatgaaatgtaggggagtacgtagaggtctatatatagcggaaatgttaagggtttgccgtt
		D36	ccatggtttcacttgatttttagtgtgactagtttttgtatgtaaggtttatgtttatgatgaaatgta
	(24)	A	
		D51	
		C26	tttatgatgaaatgtaggggagtacgtagaggtcatatatagtggaaatgttaagggtttgccgttt
	(23)	A	
		D51	
		C25	tttatgatgaaatgtaggggagtacgtagaggttatatatagtggaaatgttaagggtttgccgttt
	(24,23)	A	
		D51	
		C26,25	tttatgatgaaatgtaggggagtacgtagaggtatatatagtggaaatgttaagggtttgccgttt
	(20)	A	
		D51	
		C22	tttatgatgaaatgtaggggagtacgtaggggtctatatatagtggaaatgttaagggtttgccgttt
	(9)	A	
		D51	
		C11	tttatgatgaaatgtaggtgagtacgtagaggtctatatatagtggaaatgttaagggtttgccgttt
	(8)	A	
		D51	
		C10	tttatgatgaaatgtagaggagtacgtagaggtctatatatagtggaaatgttaagggtttgccgttt
	(9,8)	A	
		D51	
		C11,10	tttatgatgaaatgtagatgagtacgtagaggtctatatatagtggaaatgttaagggtttgccgttt
	(20,9,8)	A	
		D51	
		C22,11,10	tttatgatgaaatgtagatgagtacgtaggggtctatatatagtggaaatgttaagggtttgccgttt
	(24,23,20,9,8)	A	
		D51	
		C26,25,22,11,10	tttatgatgaaatgtagatgagtacgtaggggtatatatagtggaaatgttaagggtttgccgttt
	(24,23,20,9,8)-5′-UTR1	A	
		U1	
		C41,40,37,26,25	

## Appendix B

**Table A2:** The segregation of the T_1_ tobacco plants on selective medium.

Promoter	Deletion Variant, bp	No. T_0_ Plant	T_1_ Plants		*χ* ^2^	Segregation Ratio 3:1
			Kanamycin-Resistant	Kanamycin-Sensitive		
**pro-SmAMP1**	–425	1	372	48	41.26	NO
		2	1288	424	0.05	YES
		3	182	70	1.04	YES
	–373	1	438	91	17.16	NO
		2	429	112	5.33	NO
		3	186	59	0.11	YES
	–323	1	396	20	90.46	NO
		2	436	70	33.65	NO
		3	93	22	2.11	YES
	–307	1	371	114	0.58	YES
		2	392	22	85.57	NO
		3	350	1	114.35	NO
	–273	1	243	67	1.90	YES
		2	351	16	83.39	NO
		3	378	142	1.48	YES
	–220	1	151	53	0.10	YES
		2	432	27	89.47	NO
		3	219	2	68.43	NO
	–170	1	406	18	97.41	NO
		2	253	80	0.17	YES
		3	261	17	52.88	NO
	–102	1	302	85	1.90	YES
		2	377	114	0.83	YES
		3	456	105	11.81	NO
**pro-SmAMP2**	–438	1	302	85	1.90	YES
		2	456	105	11.81	NO
		3	377	119	0.27	YES
	–426	1	546	81	48.81	NO
		2	10	430	1241.21	NO
		3	298	97	0.04	YES
	–374	1	355	20	77.36	NO
		2	299	71	6.66	NO
		3	342	106	0.43	YES
	–324	1	353	30	60.20	NO
		2	344	132	1.89	YES
		3	358	29	63.26	NO
	–308	1	200	133	39.64	NO
		2	422	6	127.12	NO
		3	368	135	0.91	YES
	–274	1	524	45	88.65	NO
		2	69	8	8.77	NO
		3	403	144	0.51	YES
	–222	1	364	185	22.15	NO
		2	523	57	71.21	NO
		3	399	147	1.08	YES
	–172	1	730	253	0.29	YES
		2	424	137	0.10	YES
		3	615	43	119.65	NO
	–104	1	328	111	0.02	YES
		2	394	124	0.31	YES
		3	529	78	47.79	NO

For *p* ≤ 0.05 and d.f. = 1, the critical *χ*^2^ value is 3.84.

**Table A3:** The segregation of the T_2_ *Arabidopsis* plants on selective medium.

Promoter	Deletion Variant, bp	No. of T_1_ Plant	T_2_ Plants		*χ* ^2^	Segregation Ratio 3:1
			Kanamycin-Resistant	Kanamycin-Sensitive		
**pro-SmAMP1**	–425	1	509	125	9.44	NO
		2	611	131	21.35	NO
		3	988	302	1.74	YES
	–373	1	130	45	0.05	YES
		2	115	37	0.04	YES
		3	83	64	26.94	NO
		4	36	39	29.16	NO
	–323	1	264	85	0.08	YES
		2	156	9	33.62	NO
		3	95	48	5.60	NO
		4	126	87	28.52	NO
	–307	1	105	11	14.90	NO
		2	183	64	0.11	YES
		3	132	4	35.29	NO
	–273	1	151	6	37.56	NO
		2	239	0	79.67	NO
		3	302	105	0.14	YES
	–220	1	84	30	0.11	YES
	–170	1	280	35	32.41	NO
		2	70	22	0.06	YES
		3	127	43	0.01	YES
**pro-SmAMP2**	–438	1	573	194	0.04	YES
		2	475	78	35.01	NO
		3	562	37	113.19	NO
		4	713	212	2.14	YES
	–426	1	118	85	30.82	NO
		2	125	113	64.14	NO
		3	352	95	3.35	YES
	–374	1	27	30	23.21	NO
		2	65	24	0.18	YES
	–324	1	45	60	57.86	NO
		2	84	27	0.03	YES
		3	64	72	56.63	NO
	–308	1	82	26	0.05	YES
		2	53	18	0.00	YES
	–274	1	15	20	19.29	NO
		2	62	22	0.06	YES
		3	69	26	0.28	YES
		4	82	0	27.33	NO
	–222	1	338	104	0.51	YES
		2	561	106	29.51	NO
	–172	1	78	27	0.03	YES
		2	0	92	276.00	NO
		3	49	53	39.54	NO
		4	89	33	0.27	YES

For *p* ≤ 0.05 and d.f. = 1, the critical *χ*^2^ value is 3.84.
